# Exploring the Motivations and the Concerns Behind Self-Managed Medication Abortion Done by Purchasing Medication Online: Qualitative Interview Study With US Abortion Seekers Post-Roe

**DOI:** 10.2196/75780

**Published:** 2025-12-08

**Authors:** Cristina Bosco, Ege Otenen, Patrick C Shih

**Affiliations:** 1 Luddy School of Informatics, Computing, and Engineering, Indiana University Bloomington, 700 N Woodlawn Ave, Bloomington, IN, 47408, United States, 1 (812) 856-5754

**Keywords:** abortion, illicit e-pharmacy, stigma, reproductive care, self-managed abortion

## Abstract

**Background:**

As restrictive abortion laws continue to emerge in various states of the United States, pregnant individuals are turning to alternative channels to seek abortion medication, one of which is engaging in self-managed medication abortion, purchasing the medication from online websites without any medical prescriptions.

**Objective:**

This study explores the phenomenon of self-managed abortion medication sought by using online tools, by focusing mainly on the motivational factors and concerns behind such a decision, and the abortion seekers’ journey throughout this process.

**Methods:**

We conducted 20 semistructured interviews with individuals who have sought an abortion in the United States by buying medication online. The interviews occurred online, and participants were compensated for their participation. Participants were recruited from Reddit communities centered around abortion access. The interviews were audio-recorded and transcribed. The data were analyzed using a grounded theory approach.

**Results:**

The results showed that participants expressed the need for anonymity, digital and physical, when seeking an abortion and considered self-managed abortion to be more economically and time-convenient. They also discussed how much self-managed abortion can lead to isolation and anxiety because of the lack of medical support and the sole reliance on information found online. The risks, such as counterfeit medication, possible fraud, and issues of timing, associated with seeking a self-managed abortion were extensively discussed by participants.

**Conclusions:**

Our research uncovered the motivational factors behind relying on online tools to purchase abortion medication and engaging in self-managed abortion. Moreover, our research provides evidence of the importance of digital services to offer pregnant individuals a way to find abortion medication detached from their physical communities, circumventing the stigma associated with seeking an abortion and the sociocultural consequences of it. This promised anonymity is the core motivational factor that encourages pregnant individuals to prefer these systems compared to legal options. However, behind this promised anonymity, privacy and security concerns might be hidden.

## Introduction

### Background

Following the Supreme Court’s decision in *Dobbs v. Jackson* Women’s Health Organization (2022), which overturned *Roe v. Wade*, the legal landscape of abortion in the United States has become increasingly fragmented and restrictive. The geographical distribution of abortion laws now varies widely, leading to significant disparities in access to abortion services across states. While some states have preserved pre-Dobbs protections, others have enacted near-total abortion bans, with limited or no exceptions [1]. This regulatory upheaval has prompted many pregnant individuals to seek alternative means of accessing abortion care [2]. Because abortion is not banned at the federal level but restricted by state jurisdiction, some people travel to states where abortion remains legal [3]. Others have turned to self-managed abortion, that is, terminating a pregnancy outside the formal health care system [4-6].

While this practice was used even before the overturn of *Roe v. Wade*, in particular in countries and regions where clinical access to abortion is limited or prohibited [7-10], a recent study, conducted by Aiken et al [11], has provided evidence of a significant increase in self-managed abortion in the United States post-Roe.

Recent studies on the increase in self-managed abortion in the United States post-Roe have highlighted how the preference for self-managed abortion is more common among people belonging to racial minorities and underserved communities, underscoring the impact of systematic racial and economic disparities in access to abortion and reproductive care [12,13].

The practice of self-managed abortion has been widely discussed among feminist scholars, with a focus on the role of technology in enabling it. For instance, Braine [14,15] discussed how digital technology has become an infrastructure of resistance, facilitating the creation of a transnational feminist network of activist groups helping individuals in seeking a self-managed abortion. Erdman et al [16] have framed the practice of self-managed abortion in the larger context of policies of harm reduction and social change by highlighting the importance of self-managed abortion, facilitated by digital tools, as an emancipatory means for pregnant people living in restrictive contexts. Additionally, studies in the field of Feminist Human–Computer Interaction and Feminist Science and Technology Studies have widely shown how digital tools and interventions can support the achievements of Feminist-oriented goals such as body awareness and acceptance [17-19], as well as normalizing of stigmatized reproductive issues such as pregnancy loss, breastfeeding, and even abortion seeking [20-23].

### Motivations and Risks of Buying Abortion Medication From Digital Sources

Increasingly, self-managed abortions are facilitated by online platforms, particularly illicit e-pharmacies, as well as social media, in which illicit e-pharmacies are widely advertised and spread [24]. Illicit e-pharmacies refer to websites that violate legal and regulatory standards by selling unapproved or counterfeit medications or by dispensing prescription drugs without a valid prescription. They typically lack regulatory oversight, certification, or accountability [25].

While some research has explored this phenomenon, particularly in the Republic of Ireland, where abortion was illegal until recently [7,26-28], far fewer studies have examined this practice in the US context [29,30]. These US-based studies were conducted before the Dobbs decision and thus do not reflect how the recent legal changes have influenced individuals’ motivations to seek abortion medication online [29,30]. Existing research has also primarily focused on two well-known nonprofit platforms: Aid Access and Women on Web [26-31]. These services are among the most trusted for medication abortion, operating with licensed physicians and often within legal frameworks (eg, Dutch law in the case of Women on Web) [14,32]. Participants in these studies emphasized the convenience, affordability, and emotional privacy afforded by such services. In Ireland, people used Women on Web to avoid the cost, time, and stigma associated with traveling abroad for an abortion [28]. US-based users of Aid Access valued the platform’s medical credibility and affordability compared to in-clinic procedures [29,30].

Abortion stigma remains a critical factor shaping these decisions. Defined by Kumar et al [33] as “a negative attribute ascribed to women who seek to terminate a pregnancy that marks them...as inferior to ideals of womanhood,” abortion stigma is deeply intertwined with cultural norms about femininity and motherhood. It tends to be particularly intense in conservative or religious communities [34,35]. The need for anonymity, whether to avoid stigma, legal repercussions, or emotional distress, is a recurring theme in both abortion research and other domains of illicit online activity. Studies of online drug use forums, for instance, highlight how pseudonymity enables open sharing in otherwise stigmatized spaces [36,37]. Similarly, those seeking abortion medication online often value the privacy that digital platforms afford. Despite the growing prevalence of self-managed abortion, buying from illicit e-pharmacies is fraught with risk.

Fear of fraud (eg, receiving no medication or counterfeit pills), lack of medical oversight, and concerns over drug quality are major deterrents [30,38-41]. Additional dangers include privacy breaches, financial scams, and health complications arising from misinformation about dosage and administration [42,43]. These concerns are further compounded by the rampant spread of disinformation online regarding abortion methods, legality, and access.

### Misinformation and Reproductive Care

The user’s digital literacy represents an important factor in assessing the credibility of the informational sources and the credibility of the service online offering abortion medication [44]. In fact, when access to reproductive care is impeded and constrained, people will be more likely to seek reproductive health information or care using online tools and resources [45]. However, they might fall prey to misinformation and disinformation. For instance, Pagoto et al [46] illustrated how rapid changes in regulations and legislation concerning abortion access post-Roe have contributed to the epidemic of misinformation of abortion-related information online, exacerbated by the lax efforts by social media companies to mitigate the spread of it. Similarly, Acero et al [47] focused on evaluating the quality, the accuracy, and the amount of misinformation found on YouTube videos concerning abortion procedures and found that while pro-choice videos have the least amount of misinformation, antichoice videos present the highest amount of misinformation. Their study highlights the perniciousness of misinformation online on abortion access and procedures, as well as the danger of antichoice videos advancing their agenda by intentionally spreading misinformation. Their findings are not a novel consequence of the post-Roe abortion landscape, as even when abortion was constitutionally protected, antichoice organizations often masquerade as legitimate sources to mislead users and delay or block access to abortion care [48]. These studies show how misinformation on abortion access and reproductive care presents a serious concern for women seeking an abortion, as it might seriously impact the opportunity for women coming from lower socioeconomic status and racial minorities to safely engage in self-managed abortion done using online tools [12,13].

### Reproductive Care and Digital Surveillance

Concerning the phenomenon of buying abortion medication online, one final aspect concerning privacy and data protection must be carefully addressed. While digital tools might promise their users a new form of anonymity, they might introduce new severe privacy vulnerabilities and concerns. Studies in the domain of the intersection between Fem-Tech systems and cybersecurity have shown the privacy concerns of using these systems to manage reproductive health.

For instance, Cao et al [49] have shown that users often lack awareness of the data protection practices of these technologies, failing to understand what kind of data are collected, how their data are stored, and whether or not their data can be sold to third parties. Additionally, many Fem-Tech solutions are characterized by privacy policies that are legally dense, vague, and extremely hard for users, who are not experts in cybersecurity, to navigate and make sense of [50,51].

The possibility of digital surveillance conducted through the use of Fem-Techs represents a severe issue, in particular, in the current political and social climate in the United States, in which digital intimate data, such as period tracking or fertility tracking collected by Fem-Techs, could be used for criminal evidence [52] against the users themselves. This can result in what Ulrich and Fowler [53] describe as “Fem-Tech Dystopia,” a society in which the technologies designed to improve women’s agency and control over their body and foster bodily autonomy become a tool for surveilling and limiting those same women’s bodies and choices. In this “Fem-Tech Dystopia,” women are not only surveilled through their Fem-Tech devices but privacy risks extend to the use of any digital tools (eg, browsing history, credit card payments) [54], showing that in a state of capitalism of surveillance, nobody can escape digital mass surveillance [55].

### The Goal Study

While studies to date have offered important insights into self-managed abortion, their focus on reputable providers like Aid Access and Women on Web limits our understanding of the broader and increasingly complex landscape of online abortion medication. Many individuals, especially those unfamiliar with or unable to access these services, turn to lesser-known or unregulated websites, navigating a digital marketplace that blends trustworthy, fraudulent, and dangerous sources.

This study aims to expand on prior research on this topic by investigating the experience of individuals living in the United States who sought a self-managed abortion online, through illicit e-pharmacies, in the wake of the Dobbs decision. The focus of this paper is on understanding their motivations, their perceived risks, and their digital practices while seeking a self-managed abortion using online tools.

Based upon prior literature on this topic, we hypothesize that people engaging in the practice of self-managed medication abortion, by seeking medication online, are motivated by the need to avoid abortion stigma and to circumvent the restrictions in place.

## Methods

### Recruitment

Participants were recruited solely on Reddit through a self-selection sampling strategy. A recruitment post (see Multimedia Appendix 1) was shared on three abortion-related subreddits: r/abortion, r/DIYabortion, and r/birthcontrol. Interested users were invited to message the first author directly. Additionally, we also contacted users directly who have shared their abortion stories on Reddit by sending a private message to them. A total of 23 users contacted the first author expressing interest in the study. None of the people who were sent private messages replied to the researcher. Follow-up messages were sent, and 20 participants confirmed continued interest and participated; 3 did not reply. Eligibility criteria required participants to have had a personal experience seeking an abortion using self-managed methods enabled by digital tools and to be over 18 years old.

### Data Collection

All interviews were conducted by the first author, a PhD student in Human–Computer Interaction with training in qualitative methods, who identified as a woman. Prior to the interview, participants were informed that the research concerned their experience seeking a self-managed abortion using online tools. Before each interview, the first author, who conducted the interviews, introduced herself to the participant by stating her name and her occupation.

The methodology chosen for this study was the use of semistructured interviews. The interview guide was structured around 5 different themes: (1) demographic information (age, gender, sex, whether or not they had medical insurance, and the state they were living in), (2) the process of decision-making and motivation, (3) information seeking around seeking an abortion, the experience of seeking an abortion using digital tools, (4) logistics and safety concerns, and finally (5) the social and emotional context. The full interview guide can be seen in Multimedia Appendix 2.

The interviews were conducted on Zoom, in October 2023, wherein only the first author and the participant were present. The interviews lasted between 20 and 35 minutes, and they were audio-recorded. The participants were compensated with a US $10 Amazon gift card.

We considered data saturation to have been reached around the 18th interview, as no new themes were emerging at that point. However, we proceeded with two additional interviews that had already been scheduled with participants. No field notes were taken during this process.

To each participant, a unique code and a pseudonym were assigned. Each interview was then automatically transcribed using the Otter.ai tool [56], and all transcripts were anonymized. The audio recordings were subsequently deleted. No interview was repeated. Given the efforts to protect participants’ anonymity and privacy as much as possible and limit the communication between the research team and the participants, the transcripts were not returned to participants after they were obtained.

### Data Analysis

Data analysis was conducted by the 3 researchers involved in this study, and it was conducted using Taguette [57], a qualitative data analysis tool, which enabled the research team to collaboratively code and manage the dataset. While the interview guide was informed by prior research, the analysis followed a grounded theory approach, allowing key themes to emerge inductively from the data [58].

Each member of the research team first read through all the transcripts obtained to familiarize themselves with the dataset. Then, each member, individually, identified codes that were recurring in the dataset and created their individual codebook. This codebook was then shared with the other 2 researchers in several group sessions, wherein, at the end, a shared codebook, built upon individual codebooks, was created. This shared codebook was then used to reanalyze the data and look for repeated patterns in the dataset. From the aggregation of several codes, 4 unique themes were created. The final coding structure consisted of 4 main themes: (1) a strong desire for anonymity, both physical and digital, is fulfilled; (2) self-managed abortion, conducted by purchasing medication online, is cost-effective and convenient; (3) digital and physical anonymity can lead to isolation and feelings of loneliness; and (4) the promised convenience can often hide negative health consequences.

We also found a set of subthemes nested under each, reflecting recurring patterns in the data. Given the effort to protect participants’ anonymity and privacy and limit the communication between them and the research team, participants were not asked to validate the findings.

### Positionality

Openly and critically reflecting on the authors’ (personal) positionalities helps contextualize the shaping of research questions, analytical perspectives, and the interpretation of findings, as well as enhancing the transparency of the research [59]. In this paper, we consider positionality to be especially important given the historical underrepresentation of women’s health in research. All of the researchers live in the United States, in a state where access to abortion has become illegal after the overturn of *Roe v. Wade*. This drastic change in abortion law in their state encouraged them to conduct research in this sensitive domain. Additionally, all of the researchers are strongly pro-choice and believe in enhancing reproductive justice for all [60], which shaped the research question by focusing on understanding, to assist people to manage their reproductive health and care, when denied by their state. Finally, the research team has extensive experience researching in the field of health informatics and designing technologies for marginalized or stigmatized populations, using qualitative methods such as interviews, focus groups, and co-design methodologies.

### Ethical Considerations

We received ethical approval from Indiana University (ethics board approval number 17171); however, given the sensitive nature of the topic, stringent measures were implemented to ensure the privacy and confidentiality of the participants. Before the interviews, participants were explicitly instructed not to disclose their real identities to the researchers and were advised against enabling their computer cameras. Participants provided informed consent prior to being included in the study.We intentionally refrained from collecting personal information such as participants’ race, employment status, education, and sexuality. This decision was made to prioritize and safeguard the privacy and confidentiality of our participants. Throughout the interviews, participants were consistently reminded to refrain from sharing their real names. After each interview, we create alternative email addresses for the participants, which were subsequently used to distribute the Amazon gift cards (US $10). Furthermore, participants were encouraged to delete any emails exchanged with the researcher for scheduling purposes or containing information related to the study. For protecting participants’ privacy, each one of them was given a pseudonym throughout all data analysis. Moreover, the quotes used throughout the papers have been modified from their original versions to make it sufficiently impossible to link a specific quote to a specific participant. Additionally, in order to safeguard the participants’ information, we sought and obtained a certificate of confidentiality from the National Institutes of Health.

## Results

### User Statistics

As shown in Table 1, participants’ age ranged from 22 to 32 years, with 20 people who were assigned female at birth and identified as women. Most of the participants (15, 80%) came from a state where abortion is legal, while few of them (5, 25%) came from a state where it is illegal. In addition to that, half of them (10, 50%) had some form of medical insurance, while others (10, 50%) did not have any health insurance. All of them reported that they had no prescription from a doctor when they sought and bought abortion medication online. Our research has aimed to deepen the current understanding of the complexity involved in seeking abortion medication from illicit e-pharmacies, focusing on the motivations behind such a choice. We have identified 4 main themes, which will be discussed in the following sections.

**Table 1.** Aggregate summary of participants’ demographics.

Characteristic	Value
Total participants, n	20
Age range (y)	22‐32
Gender, n (%)	
Women	20 (100)
Sex assigned at birth, n (%)	
Female	20 (100)
States represented, n (%)	
California	5 (25)
New York	6 (30)
Texas	3 (15)
New Jersey	2 (10)
Indiana	1 (5)
Colorado	1 (5)
Nevada	1 (5)
Wisconsin	1 (5)
Abortion legality in state (at time of study), n (%)	
Legal	15 (75)
Illegal	5 (20)
Medical insurance coverage, n (%)	
Yes	10 (50)
No	10 (50)
Had a prescription for abortion medication, n (%)	
No	20 (100)

### A Strong Desire for Anonymity: Both Physical and Digital Is Fulfilled

The first most relevant theme identified by the interview was the desire for anonymity. An overview of all the different forms of anonymity can be seen in Table 2. All of the participants, when asked why they chose to opt for an illicit e-pharmacy to obtain medication abortion, claimed that obtaining the medication online instead of going to a physical pharmacy allowed them to hide their true identity and granted them the possibility to seek an abortion in complete anonymity. In their opinion, two forms of anonymity could be achieved using online websites: digital anonymity and physical anonymity.

**Table 2.** Forms and functions of anonymity in seeking abortion medication via illicit e-pharmacies.

Types of anonymity	Description and participant insights
Digital anonymity	Participants avoided sharing personal data (eg, name, address, card info) by using fake names, public delivery spots, and anonymous payment methods.
Physical anonymity	Online services removed face-to-face interactions, helping participants avoid being recognized or exposed.
Stigma avoidance	Participants feared judgment from family, community, and professionals. Anonymity helped shield them from this stigma.
Circumventing legal barriers	In states with abortion restrictions, anonymity allowed participants to access care without legal interference.

Digital anonymity was ensured by purchasing online, as while doing so, the user is not asked to share sensitive information. Some participants, as P15, reported that they received the abortion medication using a fake name and providing a shipping address, a public location.

*I didn’t put my home address on the website, because I didn’t feel safe using my address. I just gave a public address, like a park, and somebody went to get it for me.*

[P15]

This method granted them digital anonymity and avoided them being linked with the need for seeking an abortion. It is worth noticing that none of our participants mentioned the fact that websites can potentially track their users and collect sensitive information about them, nor did any of them mention the use of strategies to protect their identity online, probably believing that not providing private information could be enough to protect their digital persona.

Concerning physical anonymity, our participants believed that websites, such as illicit e-pharmacies, represented the best strategy to protect their physical anonymity as they grant no face-to-face interaction with any medical personnel or pharmacists.

*Most of these websites don’t require a prescription. You can just go for the one that you think best works for you, without leaving your home, without anyone seeing you at the store or the doctor’s office.*

[P8]

Avoiding face-to-face interactions with medical professionals can also help participants avoid the fear of being judged for seeking an abortion and shield them from the stigma

*I was concerned that I could have been criticized by a medical practitioner, if they had found out I sought an abortion. I could have been criticized in a very bad way and I was gonna lose my self-esteem, and I would have not been able to talk to another practitioner again.*

[P5]

Abortion stigma is not only related to medical personnel; rather, it extends to family members, community members, and significant others. For some participants, the physical anonymity offered by online websites represented a solution to hide their intentions to seek an abortion from their loved ones.

*My mom doesn’t know about this, because I haven’t told her anything. I was afraid that she might judge me. If she found out, I would feel embarrassed about what I was doing. I was concerned: what if she finds out? How is she going to react? And what is she going to say about me? It just worries me too much.*

[P5]

Finally, the physical anonymity offered by digital technology grants the opportunity for pregnant individuals, living in a state wherein abortion is illegal, to access their reproductive rights. When seeking medication using formal channels has become impossible, digital technology can offer them the chance to manage their reproductive health without their state’s awareness or involvement.

*The new law that has been put in place in Texas is limiting and subjecting most of these women because abortion can only be done legally on women, only if you are about to lose your life. When you’re not losing your life, or there are no complications with your pregnancy, you are not allowed to terminate a pregnancy. It is better to confine yourself to online services that will deliver the package to you at your house through the mail.*

[P9]

This section has provided evidence of the importance of anonymity for pregnant individuals seeking medication abortion online, emphasizing how digitally mediated abortion is perceived as the best strategy available to seek an abortion in digital and physical anonymity.

### Self-Managed Abortion, Conducted by Purchasing Medication Online, Is Cost-Effective and Convenient

Digitally mediated self-managed abortion offered participants the opportunity to seek an abortion at cheaper costs, see Table 3.

**Table 3.** Affordability and convenience in digitally mediated abortion.

Theme	Description and participant insights
Lower cost	Online medication (US $100‐180) was significantly cheaper than institutional options (US $500+), especially for uninsured participants.
No doctor visit needed	Online access eliminated the need for costly or inaccessible doctor appointments.
Familiarity with e-Commerce	Online abortion felt similar to routine online shopping, reinforcing normalcy and ease.
No transportation required	Online access avoided long or difficult trips to clinics or pharmacies, especially in rural areas.

Indeed, seeking medication abortion online can be significantly cheaper than either traveling to another state for an abortion (as in the case of participants from Texas) or purchasing medication abortion without medical insurance. The cost of abortion medication obtained through an illicit e-pharmacy (US $100‐180) is considerably lower than the cost of medication acquired through institutional health care facilities without health insurance (roughly US $500). Given that half of our participants reported not having medical insurance coverage at the time of the study, they found it more economically viable to seek abortion medication online

*When the prices are very outrageous, and you look at the prices and you will be wondering if there are other options. Then I went online, checked out the price, and I found what I wanted.*

[P7]

For some participants, convenience was also linked to the ability to bypass even the need to visit a doctor, which can be an additional cost for those without medical insurance, as the medication can be accessed and obtained online without a prescription.

*I would say it is very convenient: it saves time as I will not have to physically see a doctor, and I can access the medication using my phone.*

[P12]

In this context, buying medication for abortion online is viewed as similar to any other online purchases. Just as one can buy anything online (from clothes to furniture), it is not unthinkable to purchase abortion medication in the same way. Buying medication for abortion online does not represent an exception to a person’s daily purchasing routine.

*It’s very convenient for me to just use my phone. The world is technically global now, you know? You don’t need to be, go in-store to get whatever you want. I’m so used to getting things online. This wasn’t an exception.*

[P14]

Additionally, using digital technology avoids the need for transportation to a pharmacy or doctor’s office, which, for people living in rural and underserved suburban areas, might represent a significant challenge.

*Since it is a suburban place, we didn’t have enough pharmacies. Online, you just have to stay at your home and pay. With the delivery, you get it, you don’t have to drive anywhere or get a bus anywhere.*

[P15]

Therefore, for our participants, seeking medication abortion online represents a convenient choice both economically, as it is significantly cheaper than formal channels, and practically, as it spares them the burden of transportation to reach a pharmacy or doctor’s office.

### Digital and Physical Anonymity Can Lead to Isolation and a Feeling of Loneliness

The anonymity offered by the use of digital technology, while enabling individuals to hide their intentions and obtain an abortion in anonymous terms, can contribute to creating a sense of isolation during the process, see Table 4.

**Table 4.** Isolation and lack of support in self-managed digital abortion.

Theme	Description and participant insights
Emotional isolation	Participants felt alone and anxious, with no one to turn to for reassurance or guidance.
Overwhelmed by options	Uncertainty around which websites were legitimate or safe caused stress.
Lack of medical support	Participants often bypassed medical consultations, intensifying the emotional and health risks.
Inadequate information	Websites gave vague instructions, leaving users uncertain about the medication’s effects.
Turning to online communities	Participants sought peer support on Reddit and women’s health forums.
Fear of legal repercussions	The anonymity that protected them also discouraged seeking medical help if complications arose.

Seeking an abortion is a unique experience, and when done through digital means for the sake of remaining anonymous, individuals can find themselves alone in managing this complex task.

*I remember during my first search, I was just full of anxiety because I wasn’t sure about the decision I was making. I was terrified that I was alone. I didn’t have any clues. I was just shocked and full of anxiety.*

[P10]

This feeling of isolation becomes even more stressful as individuals navigate the complexity of dealing with multiple online services offering medication abortion. They often do not know among those websites which one is reliable, which one is fraudulent, and which one is safe.

*The challenge is: which website would I use? Where do I go? How do I use this website? How am I sure I’m gonna get this pill? And there are so many things on my mind. At the same time, I also needed to get this pill because I needed to get rid of the pregnancy as soon as possible.*

[P8]

Left without professional guidance in this complex landscape of options and information, participants had to find their own ways to assess the reliability of the information and the options found online. In doing so, they utilized similar strategies as they would for any other online platform selling goods online: they searched for online reviews about the online platform as a way to judge its reliability.

*There were reviews, there were recommendations, a lot of things were put on the internet to enable them to gain people’s trust. I think that was what also made me give them my trust about their service.*

[P14]

On a similar vein, some searched for forms of official and institutional licenses of the platform

*They just said they have the approval of the FDA to operate. So I just said: okay, I will use their service then.*

[P9]

The use of the reviews and the presence of official and institutional licenses were considered as indicators of trustworthiness of the websites, signaling their reliability. Some looked for the presence of a customer service to guarantee the credibility of the online platform.

*I felt like this website was safer because you could easily file complaints and you get feedback from them, or you can get advice or recommendations.*

[P17]

Yet, the use of all these assessment strategies does not seem to be enough to protect the participants, as some of them claimed to be victims of scams as they placed orders that never arrived. This happened in websites that met all the criteria presented above: good reviews, FDA license, and customer service, showing the evident limitations of adopting these tools when assessing an online platform’s credibility.

*I’ve tried several strategies, and then to my disappointment have been scammed: the pills have never been brought to me. I used a particular website that got a recommendation from a friend too. They’ve never arrived.*

[P4]

While some e-pharmacies offer the option to consult with a medical doctor before obtaining medication, some women prefer to maintain their anonymity and choose to reject this option. Moreover, going through the entire process of seeking an abortion without any medical or professional help, as well as with the time pressure to initiate the abortion process, adds to the emotional burden.

*It was risky, because I was not given the prescription by a professional. So it was risky, because I used the exact medication without knowing the risks to my health. I was just taking them based on recommendations found online, not based on prescriptions.*

[P17]

As most people search for illicit e-pharmacies selling medication abortion, and since this search is done alone without medical guidance, they must rely on the information provided by these websites themselves. Unfortunately, this information is often not accurate or complete enough to guide them properly.

*On the website, it was just written the price and how to use it. They (the website) just told me that I was supposed to read the prescriptions there. So they did not answer my question the way I wanted. I wanted them to explain it to me clearly.[...]They just told me that I had to follow the instructions printed on the paper. That’s what was on the pills inside. I expected them to give a good explanation, but nobody gave it to me.*

[P15]

In some cases, as information on the illicit e-pharmacies is inappropriate, and they lack medical guidance, women turn to abortion-specific Reddit subcommunities wherein women’s health is discussed by women.

*I searched for women’s health groups. I search for pregnant women, for basically mostly women and help groups there. Why? Because of women’s health, they care about the health, welfare of women, and will want anything that will best be helpful to the women. They are quite anonymous; your identity won’t be revealed. They just have this private conversation, you just have to register for the group, have access, and there are just a few women that are there that will discuss the websites recommended to you.*

[P13]

This isolation can also reduce their willingness to seek medical help, if something goes wrong. Participants worried that if the medication was not effective and they had to go to a hospital, medical providers might recognize the symptoms of an illegal abortion, and they could face legal consequences.

*But, I may have to face the authorities for taking something illegal, something that I shouldn’t have taken at the moment, something that is not readily available to me. So I may have to answer certain questions like: how did you get it? Where did you get it? And all of that.*

[P4]

Thus, it could be concluded that while digital and physical anonymity provided by online platforms can protect pregnant individuals’ privacy and shield them from abortion stigma, it can also lead to significant feelings of isolation and loneliness.

### This Promised Convenience Can Often Hide Negative Health Consequences

Seeking medication abortion solely relying on digital technology can fulfill the desire for convenience; however, it might often hide severe negative consequences, see Table 5. Participants were very aware of these risks and considered them their main concerns. One risk mentioned by participants is the fear of being defrauded:

*A whole lot of women are rushing online to get this set of pills, so those people should be careful, because a long list of websites are not legit.*

[P14]

**Table 5.** Negative consequences of self-managed abortion done using online tools.

Theme	Description and participant insights
Fear of fraud	Participants were aware of the risk of fraud and took precautions such as asking friends for advice and checking reviews.
Consequences of fraud	Fraud resulted in payments made but no medication received, leading to frustration and financial loss.
Timing issues	Participants often bypassed medical consultations, intensifying the emotional and health risks.
Lack of legitimacy	Participants worried whether the medication was the right drug or genuine, highlighting the absence of guarantees.
Concerns about effectiveness	Concerns over the quality of pills, such as being counterfeit or damaged, weighed heavily on participants.
Urgency over risk	Despite concerns, participants felt the urgency of their situation outweighed the potential risks.

In case of online fraud, this results in making a payment and waiting for a package that never arrives. One participant, in particular, recalled her experience, being a victim of online fraud and attempting to contact the website seeking answers and never receiving either the medication or her money back.

*The pills have not arrived yet, and to make matters worse, there is no feedback from their customer service. I have sent a couple of emails, but I got no response. Normally, if there was a delay of some sort, there would have been some feedback or some response to the customers. But there’s been nothing. Ever since I contacted them, and I made payments, it’s been like a dead end.*

[P4]

Timing is also a critical aspect in predicting the medication’s efficacy: the use of misoprostol and mifepristone is recommended up to 12 weeks into a pregnancy. Thus, the fear of waiting too long for medication bought online was discussed by some participants as one of their fears.

*Honestly, I believe that I’m running out of time because I know Mifepriston and Misoprostol do not work forever. I have not done a lot of research, but I know that at some point, they are not going to be effective.*

[P16]

In the case of these participants, being fraud does not only refer to losing money; rather, it extends to waiting for a package which never arrives, ultimately missing the possibility of using abortion medication as an effective and safe method to seek an abortion.

Additionally, purchasing medication online outside of a formal setting comes with not having a guarantee of the legitimacy of the medication itself. Indeed, while at the pharmacy, a customer can be guaranteed that they are buying the medication they are looking for, the same cannot always be applied to illicit e-pharmacies selling medication for abortion.

*I was concerned that this was not the right medication for me. I was looking at the package and wondering, what if they are not the right drugs? And if they are not the right drugs, then which ones would they be? I was just told about these. And I tried them out, regardless of the risks.*

[P11]

Hence, a big problem is assessing the quality of the medications once they arrive at the buyer. Some of the participants interviewed worried about the effectiveness of the pills bought using these services. They question whether those medications were faked, cloned, or damaged in any way, wondering also about the impact that those medications could have on their health.

*I have concerns, great concerns, because this is my health we’re talking about, most of these pills are not original. Some of them are poorly made, like counterfeit or something.*

[P4]

However, given the urgency and the uniqueness of the situation, participants still decide to rely on digital systems, preferring them to physical ones.

*I had great concerns, but at the moment, I needed it. So I am, I just had to do what was what they wanted me to do, because I also was on the other side needing their service.*

[P9]

This section shows that women are aware of the unique risks associated with seeking an abortion medication online using illicit e-pharmacies. Thus, their choice to prefer online service is not motivated by a lack of understanding of the risks associated with it; rather, it is motivated by the perceived benefits of using illicit e-pharmacies, which outweigh the perceived risks. By pondering the negative with the positive aspects, they make a conscious choice and prefer using digital services.

## Discussion

### Principal Results

This study sought to understand the phenomenon of purchasing abortion medication through online tools, especially in the wake of the post-Dobbs decision. A central objective was to explore the reasons why digital technologies are used by pregnant individuals to seek abortion medication and their experiences using online tools.

Our findings revealed that a major motivation behind the use of online tools to seek abortion medication was the desire for using a system that could offer participants a sense of complete anonymity. Participants viewed these tools as methods where they could avoid interactions with medical providers, family, community, and partners. This need for privacy was strongly linked to the stigma surrounding abortion, as well as concerns about legal consequences and personal safety. Even in states where abortion remains legal and telehealth options are available (eg, New Jersey and California), some participants still chose illicit sources due to perceived or actual barriers, including hostile health care professionals, concerns about insurance confidentiality, and low literacy regarding legal abortion options.

Despite being aware of the risks involved, such as counterfeit medication, fraud, and potential privacy breaches, participants often prioritized anonymity over safety. This reflects a broader strategy of minimizing intermediaries in the abortion process: removing health care providers and insurance companies from the equation to ensure discretion.

However, while participants expressed the need for digital systems allowing them complete physical anonymity, they do not seem to be aware of additional potential privacy issues linked to the tools they were trusting and that such systems may be collecting sensitive data about their users, such as data location, credit card details, and IP addresses. These sensitive data could be used against them, especially in states where abortion is criminalized. As a result, the perceived anonymity enabled by these platforms may, in fact, compromise users’ control and autonomy, turning these technologies into tools of surveillance and control.

### Limitations

Our study is subject to several noteworthy limitations. First, our sample consists of self-selected individuals who willingly shared their experiences of purchasing abortion pills online with our research team. This may introduce a potential bias, as those who choose to participate may not be representative of the broader population. Furthermore, the recruitment of participants was conducted mainly through Reddit, which may further limit our findings’ generalizability. Future research could attempt to overcome the limitations mentioned above by attempting to achieve different population subsets, by employing diverse recruiting strategies.

Second, only 4 out of 20 participants were living in states where abortion is illegal. This limited representation of people living in restrictive states impacts the transferability of our findings to people experiencing legal restrictions concerning abortion access. Future works should have the goal to recruit participants from states where abortion is illegal, to fully grasp the impact of legal restrictions on their motivations to practice self-managed abortion using online tools.

Third, a significant portion of our interviews relied on participants’ recollections of past experiences, often dating back to a distant period (eg, 3, 4 mo before the interview). This retrospective nature of the data collection process could introduce inaccuracies in participants’ recall of their experience.

Moreover, while we gathered demographic information such as age and gender, we did not collect any information concerning participants’ digital literacy and digital skills. Given the negative impact of misinformation found online on this sensitive topic [61], we believe that future iterations of this study should expand by addressing how users’ digital skills and literacy might mediate the experience of seeking a self-managed abortion using online tools.

Finally, we intentionally refrained from collecting participants’ personal information (such as race, education, sexuality) to protect participants’ privacy. We acknowledge that the collection of such information could have potentially enriched our understanding of the phenomenon under investigation by providing insights into the influence of various sociocultural factors on participants’ decision-making processes and access to reproductive health care [12,13,62-64]. Future work must attempt to overcome this limitation by finding ways to incorporate factors such as race, sexuality, and socioeconomic status into the analysis of the phenomenon, while safeguarding the privacy and anonymity of participants.

### Comparison With Prior Work

The findings align with previous studies documenting pregnant individuals’ preference for online abortion services due to the desire for anonymity and avoidance of stigma [26,28,30]. Similarly, prior research on online drug markets shows that perceived anonymity online can offer a form of refuge from societal judgment and stigma avoidance [36,37]. This study builds on that foundation by emphasizing how anonymity is not just a preference but often a necessity driven by fear of legal and social consequences and emphasizing how participants believe that online tools and platforms selling abortion medication can be the only solution to protect their anonymity.

While using digital tools for reproductive health can provide anonymity from one’s community, loved ones, and even health care professionals, it also introduces serious risks. A major danger is the prevalence of misinformation about reproductive care online, which can negatively impact a person’s ability to safely and effectively self-manage an abortion. If online resources are the only ones available, misinformation becomes a critical concern. While misinformation about abortion access and legality was prevalent online, even before the overturn of *Roe v. Wade* [46,48,61], in the current post-Dobbs context, confusion and misinformation concerning abortion access have become even more pronounced [47], impacting more negatively those seeking a self-managed abortion using exclusively, or largely, online tools, resources, and platforms. While digital literacy might partially protect some of them from being negatively impacted by this misinformation, people seeking an abortion who have low socioeconomic status and low digital literacy might be even more harmed by the presence of online misinformation, which can negatively affect their ability to manage their reproductive health [12,13]. Additionally, even those who might present a high level of digital literacy might fall prey to narratives and illusions concerning the anonymity promised by those online tools.

Indeed, our study revealed that the participants navigating stigma within the health care system [65] are also actively trying to circumvent the abortion stigma coming from their immediate communities, families, and loved ones. This finding shows that abortion stigma is spread among different communities, and people might perceive it even in relation to their closest family or community members [23], preventing them from accessing reproductive care in formal health care settings.

Additionally, even in states where abortion was legally protected and accessible, participants still preferred to use online tools as a way to remove all intermediaries (eg, doctors, insurance providers) from the experience of seeking an abortion, a strategy for self-protection and control. For them, the most accessible way to do this is by using online tools and platforms. They believe that operating online and relying on these tools will grant them the anonymity they need and leave no tangible or intangible traces. However, as discussed by Wu and Goldsmith’s [66] critique of disintermediation online, the idea that the internet can function as a shortcut for removing intermediaries proves to be inaccurate, as digital systems often tend to replace visible, accountable actors with invisible and less accountable ones.

In the context of digital abortion access, this shift may prove especially dangerous, as users are often unaware of the new, invisible intermediaries introduced by these systems. While traditional intermediaries such as doctors, nurses, or insurance providers are familiar and recognizable, digital infrastructures obscure the presence of data brokers, tracking technologies, and algorithmic systems that quietly mediate users’ interactions. Our study supports this understanding by providing empirical evidence that the removal of physical and well-known intermediaries is one of the key motivations for seeking an abortion using online tools. Our participants evidently valued anonymity, physical and digital, above all the other aspects (eg, the fear of fraud, the fear of counterfeit medication); however, they might not have been fully aware that using these technologies could expose them to significant privacy and security risks. By echoing a long line of research on privacy and security risks and Fem-Tech [49,50,52,54,67,68], and the definition of *FemTechno Dystopia* [53], we could argue that the same digital technologies designed to enable the user to take control and care of their bodies might be the same technologies exposing the user to privacy and security concerns.

To sum up, this study shows the often contradictory nature of digital technologies and their impact on women’s path to liberation, as proposed by Wajcman [69]. On the one hand, digital technology can provide women with the necessary tools to free themselves from external impositions and control, allowing them to circumvent the social stigma associated with seeking an abortion. On the other hand, it can create new systems of oppression and control that might undermine women’s pursuit of bodily autonomy and self-determination. Digital tools selling abortion medication fit squarely into this space: they provide women with the opportunity to seek an abortion outside a formal health care context, sparing them from stigma and protecting them from their immediate community and allowing them to manage their reproductive health in places where it is no longer available. However, they might also expose women to invisible and unknown security and privacy risks without their consent or awareness.

### Conclusions

This study demonstrates the importance of attending not only to access but to how access is mediated. The preference for illicit e-pharmacies is not simply a matter of convenience; it reflects a deeper mistrust in medical institutions, legal systems, and digital infrastructures. Designers and policymakers need to consider how systems that claim to empower users may also expose them to new risks. This is particularly crucial in the reproductive health space, where technological mediation can both protect and endanger users. Future research might investigate how to design abortion access technologies that ensure real, not just perceived, anonymity, while building trust and safety into their infrastructures. Ultimately, this study urges caution in framing digital abortion seeking as an inherently emancipatory practice. While these technologies can provide agency, they also open the door to new forms of surveillance and control, particularly when mediated by unregulated or exploitative platforms.

## Supplementary material

10.2196/75780 Multimedia Appendix 1 The recruiting messages used for participants' recruitment.

10.2196/75780 Multimedia Appendix 2 The interview guide used in this study.
