# Supplementary material for: Exploring the Motivations and the Concerns Behind Self-Managed Medication Abortion Done by Purchasing Medication Online: Qualitative Interview Study With US Abortion Seekers Post-Roe
Source: J Med Internet Res. 2025 Dec 8;27:e75780. doi: 10.2196/75780 (PMC12686335; doi:10.2196/75780)
Supplement: Multimedia Appendix 1 [file jmir-v27-e75780-s001.docx]

Appendix A: Recruitment messages

## Examples of recruitment messages:

### Social media post + caption

Our research team at Indiana University is looking for people who are 18+ and have experience buying abortion pills from e-pharmacies to participate in a study on people’s experience with e-pharmacies. Participants will be asked to take part in an interview and will be compensated with an Amazon gift card.

To learn more and/or take part, contact one of our researchers:

Researchers’ email address

### Reaching out to people who have already shared their experience on using e-pharmacy on social media publicly

Hello! My name is First Author . I'm a Phd Student at Indiana University, working in the [ProHealth Research Lab](https://luddy.indiana.edu/research/research-areas/proactive-health.html). Our principal investigator is Third Author in the Luddy School of Informatics, Computing, & Engineering.

Our team is recruiting participants for 30 minutes to 60 minutes- long interview on their experience of using e-pharmacies to buy abortion pills. We are interested in how these new services can support and help people. I am reaching out to you because you have already shared your experience on social media publicly.

If you are interested, just send me an email at the following address:

Researchers’s email

Feel free to pass my contact to anyone else you might think might be interested in participating

and please reach out with any questions!

Thank You & Best Wishes,
