# Supplementary material for: Exploring the Motivations and the Concerns Behind Self-Managed Medication Abortion Done by Purchasing Medication Online: Qualitative Interview Study With US Abortion Seekers Post-Roe
Source: J Med Internet Res. 2025 Dec 8;27:e75780. doi: 10.2196/75780 (PMC12686335; doi:10.2196/75780)
Supplement: Multimedia Appendix 2 [file jmir-v27-e75780-s002.docx]

Appendix B: Interview Guide

**Theme 1: Demographic information**

- Age, Gender, Sex,
- Do you have health insurance?
- Which state do you live in?

**Theme 2: the process of decision-making and motivation**

- When was the last time you used e-pharmacies to buy abortion pills?
- Could you walk me through what you did?
- Could I ask you what motivated you to seek abortion pills online?
- If it is legal, why did you prefer it to consulting a physical center?
- If it is illegal, why did you prefer it to other options (travelling out of state)?

**Theme 3: information seeking around seeking an abortion**

- How did you find the specific e-pharmacy you have used?
- How do you discern accurate information from the rest?
- When you were looking for information regarding buying abortion pills online,
  - Where did you go?
  - What did you do?
  - How did you achieve it?
- Is there a source that you trust the most when it comes to e-pharmacy and abortion pills?
  - Why do you trust it more?

**Theme 4: the experience of seeking an abortion using digital tools**

- What made you trust this particular website?
- Was there anything specific that made you think it was reliable enough?
- Do you remember, by any chance, what was your first impression when seeing it for the first time?
- Which kind of information was provided by the website/app?
- Did you find the information provided on the website complete/exhaustive enough?
- Was there something that you wanted to know that was not present in the website?
- Did you have any issues in using the website? If so, which ones?
- How did you choose the pill to buy?
- How did you pay?

**Theme 5: Logistics & concerns**

- How was your experience with the shipping and the following delivery?
- Did you have any concerns/worries regarding this part?
- Was it possible to track the order?
- Did  you have any concerns regarding your health when taking pills brought from e pharmacies?
- What were you most worried about?

**Theme 6: social and emotional context**

- In case that something went wrong, did you have a way to look for medical help?
- After you took the pill, who or where did you go to for ask/find information after the aftermath of the abortion pill?
- Was somebody involved in this decision-making process?
- Did somebody help you with this process?
- Was somebody with you when you took them?
  - If not, why?
  - If yes, why?
